# Potentiation Physiologically Proven

**Published:** 1893-08

**Authors:** Gustav Jaeger

**Affiliations:** Stuttgart, Germany


### POTENTIATION PHYSIOLOGICALLY PROVEN.

PROF. DR. GUSTAV JAEGER, STUTTGART, GERMANY.

[Translated by B. Fincke, M. D. Continued from page 323.]

### a. THE HIGHER POTENCIES OF THE ALKALI SALTS.

As already previously stated and proved, I began to measure the higher potencies only after I had potentiated and measured all the seventeen salts until the point of indifference was passed.

At the same time, in order to save time, I made the change that I did not measure one decimal potency after the other, but always omitted one. Since the point of indifference with the different salts lay upon different potencies, next an unequalness arose in so far as the measured potency carried in one part of the salts an even number, in another part an odd number. In order to remove this circumstance, which prevented the gaining of mean values and also to establish a correspondence with the lower potencies, numbers were reckoned for the not-measured potencies by drawing the mean from the two measured numbers of the preceding and following potencies. In order, however, to express this also in the table, the reckoned numbers are printed in smaller type.

Furthermore, the reader will comprehend why I saved to me and him the laborious work of going farther than the 30th potency in seventeen different salts; from potency to potency the repetition of the same phenomenon is seen, when every new measurement brings nothing else than a confirmation of what already has been experienced dozens of times, then one says to himself: Why still more? Whoever is not content with this can go to work himself and continue it. Therefore I jumped from the 25th potency to the 30th, and, only after finishing the mentioned proving of the Kali-carbonicum according to the swallowing method, I ordered my assistants to carry the fourteen salts, the higher potencies of which I had investigated to the 1,000th potency and inserted the value of their measurement as last number in the table.

On the arrangement of table VII the following is to be said:

(*a*.) It contains as well the higher as the lower potencies of 6 Natrum, 4 Kali, and 4 Ammonium salts. By a stair-like line signifying the line of indifference, the higher potencies are separated in every group from the lower ones.

(*b*.) Besides, every salt having its series of numbers in the table, some other further series have been formed by reckoning, namely, 1. For every one of the three groups a series of mean numbers, just as it has been done in the table of the lower potencies. 2. Among every one of these three series of mean

### VII Table. Lower and Higher Potencies of 14 Alkali-salts.

(See original in German, *A. H. Z.*, p. 35.)

| | POTENCY. | 3 | 4 | 5 | 6 | 7 | 8 | 9 | 10 | 11 | 12 | 13 | 14 | 15 | 16 | 17 | 18 | 19 | 20 | 21 | 22 | 23 | 25 | 30 | 1000 | DIFFERENCE OF 3D AND 25TH POTENCIES. |
|---|---|---|---|---|---|---|---|---|---|---|---|---|---|---|---|---|---|---|---|---|---|---|---|---|---|---|
| Natrum-salts. | Mur. | — 5 | + 6 | + 7 | +23 | +25 | +28 | +31 | +34 | +35 | +36 | +44 | +53 | +50 | +47 | +49 | +52 | +61 | +71 | +69 | +67 | +69 | +71 | +84 | +84 | 76 |
| | Nitr. | —10 | + 2 | +10 | +21 | +24 | +27 | +33 | +39 | +41 | +43 | +42 | +42 | +46 | +50 | +52 | +54 | +57 | +60 | +61 | +63 | +66 | +69 | +84 | +80 | 79 |
| | Carb. | —14 | —16 | — 2 | + 5 | + 9 | +18 | +25 | +33 | +39 | +46 | +43 | +41 | +46 | +52 | +53 | +54 | +56 | +59 | +60 | +62 | +66 | +70 | +84 | +94 | 84 |
| | Sulph. | —19 | — 8 | — 4 | + 4 | + 6 | +15 | +22 | +30 | +38 | +47 | +45 | +43 | +45 | +47 | +51 | +55 | +56 | +58 | +61 | +65 | +71 | +77 | +90 | +82 | 96 |
| | Phos. | —25 | —31 | —25 | —20 | —14 | —10 | + 1 | + 7 | +10 | +11 | +12 | +10 | + 9 | +14 | +19 | +24 | +29 | +34 | +40 | +47 | +55 | +87 | +92 | +69 | 112 |
| | Brom. | —35 | —34 | —30 | —22 | —16 | —22 | —13 | — 8 | + 4 | +11 | +18 | +16 | +14 | +19 | +25 | +31 | +37 | +45 | +54 | +55 | +57 | +81 | +80 | +66 | 106 |
| | Mean of N. S. | —18 | —12 | — 6 | — 2 | + 6 | + 9 | +17 | +23 | +28 | +32 | +34 | +34 | +33 | +38 | +42 | +45 | +49 | +55 | +58 | +60 | +66 | +76 | +86 | +79 | 94 |
| | Differ'nce | | 6 | 6 | 4 | 8 | 3 | 8 | 6 | 5 | 4 | 2 | 0 | —1 | 5 | 2 | 3 | 4 | 6 | 3 | 2 | 6 | Mean Diff. 4.1 | | | |
| Kali-salts. | Jod. | — 2 | — 1 | — 3 | +11 | +17 | +28 | +24 | +41 | +45 | +50 | +52 | +55 | +60 | +66 | +64 | +63 | +66 | +69 | +72 | +75 | +80 | +85 | +92 | +95 | 87 |
| | Carb. | —37 | —19 | —16 | —20 | — 8 | + 3 | +10 | +27 | +32 | +38 | +42 | +47 | +48 | +50 | +53 | +56 | +61 | +67 | +70 | +73 | +81 | +90 | +92 | +85 | 127 |
| | Phos. | —43 | —28 | —26 | —25 | —28 | —20 | —12 | —12 | —11 | — 8 | — 7 | — 2 | + 7 | + 6 | +18 | +33 | +48 | +50 | +53 | +60 | +67 | +89 | +95 | +65 | 132 |
| | Brom. | —70 | —45 | —33 | —31 | —26 | —32 | —26 | —25 | —28 | —19 | —17 | —13 | — 7 | + 5 | +11 | +26 | +41 | +38 | +35 | +35 | +35 | +43 | +68 | +50 | 138 |
| | Mean of K. S. | —38 | —23 | —20 | —16 | —11 | — 5 | + 1 | + 8 | +10 | +15 | +18 | +22 | +27 | +32 | +37 | +47 | +54 | +56 | +58 | +61 | +66 | +77 | +87 | +74 | 125 |
| | Differ'nce | | 15 | 3 | 4 | 5 | 6 | 6 | 7 | 2 | 5 | 3 | 4 | 5 | 5 | 5 | 7 | 10 | 2 | 2 | 3 | 5 | Mean Diff. 5.2 | | | |
| Ammonium-salts. | Carb. | —50 | —36 | —36 | —24 | —37 | —21 | —12 | — 7 | + 4 | + 3 | + 9 | + 9 | +10 | +15 | +22 | +24 | +27 | +44 | +61 | +69 | +77 | +84 | +93 | +82 | 134 |
| | Mur. | —34 | —22 | —25 | —30 | —23 | —19 | —10 | — 2 | + 4 | + 8 | + 9 | +10 | +12 | +15 | +19 | +30 | +42 | +50 | +59 | +67 | +75 | +80 | +90 | +72 | 114 |
| | Phos. | —64 | —44 | —46 | —41 | —26 | —47 | —32 | —31 | —20 | —15 | —11 | —12 | —13 | —17 | —13 | — 8 | +12 | + 4 | +10 | +15 | +20 | +43 | +34 | +62 | 107 |
| | Brom. | —63 | —43 | —45 | —40 | —35 | —38 | —32 | —29 | —29 | —25 | —18 | —23 | —22 | —33 | —24 | —21 | —15 | — 8 | 0 | +14 | + 9 | +12 | +13 | — 1 | 75 |
| | Mean of A. S. | —48 | —36 | —38 | —34 | —30 | —31 | —22 | —17 | —10 | — 7 | — 3 | — 4 | — 3 | — 5 | + 1 | + 6 | +17 | +23 | +33 | +44 | +45 | +55 | +58 | | 103 |
| | Differ'nce | | 12 | —2 | 4 | 4 | —1 | 9 | 5 | 7 | 3 | 4 | —1 | 1 | —2 | 6 | 5 | 11 | 6 | 10 | 11 | 1 | Mean Diff. 4.6 | | | |
| Total Mean, | | —35 | —24 | —21 | —17 | —12 | — 9 | — 1 | + 3 | + 9 | +13 | +15 | +17 | +19 | +22 | +27 | +31 | +40 | +45 | +50 | +55 | +59 | +69 | +77 | | |
| Total Difference, . . | | | 11 | 3 | 4 | 5 | 3 | 8 | 4 | 6 | 4 | 2 | 2 | 2 | 3 | 5 | 6 | 7 | 5 | 5 | 5 | 4 | Mean Diff. 4.7 | | | |

numbers which, of course, likewise show anteriorly minus, posteriorly plus values is a further series of numbers which (with some exceptions) have no signs. They indicate the difference of potency-numbers between which they are placed; they therefore teach by how many points the neuro-analytical number has been shifted from potency to potency. The last of these difference-numbers is between the 22d and 23d potency, then with the remark "mean difference" follows in each of the three series another number which is the mean of all difference-numbers of the series. 3. The lower end of the table is formed by two horizontal series of numbers: the upper series, which shows the numbers of plus or minus signs, gives for every potency a mean neuro-analytical number, formed out of the three mean numbers of the three groups, and the lower series of numbers without sign gives the difference from potency and at the end of the mean out of these differences (see table VIII).

Let us first comprise the whole result of the table in words as short as possible.

The series of numbers teach with the most powerful language possible in this department, viz.: that of numbers, that the potentiation actually is a gradual *increase of the motor-value* of a substance, and that here something similar to temperature is concerned; a point of zero exists for every substance in a concentration which leaves the *tempo* of the *vital motions* unchanged, evidently, because the motion of the molecules has the same velocity as that of the molecules who are already in the body. On both sides of this point of zero potentiation produces the same difference as heating. If *heat* is conducted to a cold body, the *coldness decreases* and if *heat* is conducted to a *warm* body the *heat increases.* Exactly so it is with potentiation; if a substance which acts *retarding, paralyzing* upon the vital motions is potentiated, the *paralytic effect* decreases, and if the substance is potentiated, which *accelerates* the *vital motions,* the animating effect increases. Anything clearer, according to my knowledge, cannot exist. If we add to this the experiences of daily life, which I gathered in my pamphlet, the *Homœopathic Attenuation* as—*e. g.*, the increase of the animating power of wine with increasing attenuation of its *bouquet*—it is indeed incomprehensible how it is possible that on the value and essence of potentiation such a diversity of opinion can exist as is actually the case.

Holding on to the last number of the table: this says that between the 3d and 23d potency in the mean every potency augments with me the physiologico-motorial value by 4.7 points if the proving is made exactly as I did.

Let us now consider some more details of the table.

(*a.*) The series of numbers of the single salts teach that in *none* of them the changes of their motor-value is a *uniform* and *direct one*, great progresses alternate with suspensions as well as regresses, therefore the same phenomena as we have experienced with Kali-carbonicum, and as it also appeared regularly in our first measurements in the *Neural Analyse der hom. Verdünungen.* The return of this occurrence without exception testifies that this is in the nature of the thing and not in the imperfection of the method.

(*b.*) The same appearance meets us also in the three series of the mean numbers, only naturally the irregularity is here less striking than in the series of the detail because a mutual equalization takes place, and this naturally is still more the case in the series of the total mean numbers. Let us now consider them singly: with the *Natrum*-salts the highest shifting effect is 8 plus, the least 1 minus point; therefore, a difference of 9 points. With the Kali-salts, there are no retrogressions (minus-values) in the mean, the least effect is + 2, the greatest + 15; therefore, difference, 13 points. With the Ammonium-salts the irregularity is in so far the greatest as here minus values appear 4 times, the maximal difference is 14 points. Further remarks on this see below. In the series of the total mean numbers retrogressions are deficient, the maximal difference is (11–2) 9 points.

(c.) If we consider the differences of the detail series, most strikingly different is the Brom-Ammonium, the obstinacy of which toward production of indifference we have already mentioned before. Here we have up to the 30th not only 5

retrogressions, but also uncommonly large ones—*e. g.*, from the 15th to 16th potency by 11 points—so that the 16th potency sinks under the value of the 9. When communicating to a chemist, one of my former colleagues, this singular behavior of the Brom-Ammonium, he informed me that this combination embarrassed also the chemists because it was *so easily decomposed that its vapor-density could not be determined*, whilst this was not the case with the other salts by me. This was again a remarkable proof for the certainty of Neural Analysis; it had recognized with an insuperable distinctness that this salt essentially differs from all others. Besides, this was interesting by itself. We have repeatedly found cause to make a comparison of potentiation with calorification. Now here the comparison makes itself felt forcibly. The chemist explains the abnormal behavior of Brom-Ammonium by the circumstance that in determining the vapor-density the necessary heating causes its decomposition; should not the abnormal behavior in potentiation be attributed to the same cause—*i. e.*, a decomposition—and consequently the potentiation be a similar process as the heating—*i. e.*, an increase of the velocity of motion of the molecules—to which already other phenomena and considerations point forcibly? When I had my conversation with the chemist, my investigation of the salts had only progressed as far as the 30th potency. When afterward I resolved to carry all the 14 salts up to the 1,000th potency, I was naturally very eager to know how it would be with *Brom-Ammonium.* The result was perplexing: whilst the 1,000th potency in all other 13 salts gives high animating effects (see below further remarks), I obtained with Brom-Ammonium—minus 1—*i. e., nothing! Indifference!* Of course, I have repeated the measurement several times, but always with the same result: the other 13 salts gave regularly their higher animating effects, Brom-Ammonium persistently nothing and again nothing. By the way, this is one of the cases which show the total ridiculousness of the assertion of the world-wise critics: "that the numbers of Neural Analysis are products of imagination and arbitrariness." How, then, is it possible that another number appears than the one imagined, and that with persistence? The critics are incredible hair-bristling fellows.

On this matter the following is yet to be remarked: *First*, that it would not occur to me in a dream to reject the Brom-Ammonium on account of this one experiment, for this a careful, several times repeated, after-proving is necessary which, however, I leave to those who take a special interest in Brom-Ammonium, which I do not. *Second*, if this abnormal behavior of Brom-Ammonium finds its expression again and again in the most exact after-proving, it may be expected that there are also other substances which are behaving similarly and in too high potentiation become *nothing*, which *practically* and *theoretically* is of the greatest importance. *Third*, it is of course too soon before repeated after-proving to try an explanation, but it is nevertheless natural to at least think about it, and hence I came to the conclusion if Brom-Ammonium in potentiation always behaves as in this one experiment there is no other conclusion possible than that *Bromine* is *no element*. It is clear if in potentiation Brom-Ammonium is decomposed into two parts of Bromine and Ammonium, these substances during the course of potentiation are simply potentiated further, and from these a potency near the 1,000th is formed which cannot be *nothing*. Also, when the Ammonium is decomposed into omnipresent therefore impotentiable elements, nitrogen, hydrogen, and oxygen, we do not make any progress as long as the *Bromine* will remain, only when this resolves itself into omnipresent elements indifference might, respectively, would have to occur. I will make only one more remark on this: supposed the after-proving renders a different result than what I obtained viz., that the 1,000th potency of Brom-Ammonium prove just as positive as the 1,000th potency of the other salts, we stand indeed before a greater enigma, for a confusion is impossible with the vials marked on corks and labels; what, therefore, has happened? Enough! Whoever takes an interest may follow that matter up.

(*d*.) In the detail-series and in the series of the mean numbers the procedure is only from potency to potency up to the

23d potency, then follows a jump from the 23d to the 25th, a second one from 25th to 30th, finally one to the 1,000th potency. At the *first* jump only two potencies are concerned, in accordance with the quantity of the difference. In the *Natrum*-salts the difference is between the 23d and 25th 10 points, therefore something more than double as much as the mean difference between 2 neighboring potencies (4.1); in the *Kali*-salts is the same correspondence: it is about the double of 5.2; just so it is with the Ammonium-salts (10 against 4.6) and with the total difference (10 against 4.7). I mention this only because this correspondence repeating itself *four times*, can impossibly be a product of imagination or arbitrariness. At the *second* jump of the 25th to 30th potency there is a difference of 5 potencies with opposed difference of only 10 points. Here, again, it is so remarkable and testifying in a high degree for this method that this difference of 10 points is the *same* in *all* three groups of salts and then of course also in the total mean. But $\frac{10}{5}$ is only 2. Now the number 2 indeed occurs variously in the series of the difference-numbers from the 3d to 23d potency, but 5 times successively, *never;* what does this signify? How can this sudden little decrease of the potentiating effect be explained? Still more glaring this circumstance appears at the third jump from the 30th to the 1,000th potency, for here the matter stands thus: if we abstain from Brom-Ammonium, the numbers show only in 3 salts a progress of the animating effect from the 30th to the 1,000th potency, in kitchen-salt the numbers are equal and in 9 salts a retrogression has taken place. Does not this all strike in the face what we have said hitherto? In order to answer this question we must compare both series of investigation, the series of the experiments by swallowing with Kali-carbonicum, and the present experiments by inhalation with the 17 salts. The former has shown us that in the heightening of the motor-effect by potentiation *two factors* are concerned, the *intensity* and *duration* of action. Especially we saw there that in relation to the latter factor the duration we have to distinguish two phases, a *first* and an *after-*

action, that this latter gains its full developement only at the 30th potency, and, finally, that the eminent action of the 1,000th potency depends mainly upon the exceedingly long duration of an exceedingly high first-action and again of a long-lasting moderate after-action. Now all this is of no consequence in our proving of the 17 salts, because I took in all potencies only 4 decade-numbers, and then broke off the measurement. The moment of time, therefore, is in this kind of proving entirely out of consideration, and on the questions whether under continued protentiation the duration of action increases, whether after-actions occur which again are very different in height and duration, as our swallowing experiments taught, we do not get an answer at all. I also draw the attention to the difference in the two modes of measurements. The table I gives for every potency of Kali-carbonicum a whole series of numbers (from 15–33), our table VII only *one single* number. Here also the question arises; can the series of numbers which the potencies of Kali-carbonicum received in table VII in any way compare with the numbers of table I? This question cannot only be answered with "yes," but its answer is also instructive for the estimation of the two modes of ingestion of the medicines: deglutition and inhalation, therefore we shall approach the matter nearer, though not here but in a later part of this work, since I have performed still other measurements for the sake of answering this question, and cannot here communicate them as it were in parenthesis. I only say here that the so differently obtained series of numbers for the potencies of Kali-carbonicum do not contradict themselves, as will be shown later, but correspond so well, as it is at all possible in two investigations which (1) are so far apart in time, (2) are made according to different methods, and (3) were executed with two different objects—*i. e.*, with two doses of medicine potentiated at different times in different vials.

(*e.*) If we now still compare the salts with each other according to their higher potencies (it will according to the remarks sub d) recommend itself to leave out the 30th and the 1,000th potency; for since their main action and the essential of it, the long duration, finds no expression in our numbers, the comparison is also of little value; on the contrary, it recommends itself for the potencies from 3 to 25. For this purpose the last vertical column has been made which gives the differences between the numbers of the 3d and 25th potency of every salt and of the mean values of each of the three groups. We call this difference the *total potentiating effect.* These numbers teach us that also here as in the position of the point of indifference and in the behavior of the lower potencies remarkable differences exist among the various substances. If we leave aside the abnormal Brom-Ammonium a rule—though not without exception—meets us: *the less the paralytic effect of the 3d potency, the less the total potentiating effect; and the greater the first, the greater the latter.* Let us examine this closer. In the Natrum-salts the ascending series of the total-effect-numbers from above downward corresponds with the equal behavior of the numbers of the 3d potency, but with the exception of *Brom-Natrium;* this retrogresses with the number 106. In the *Kali*-salts the rule is verified completely and contrary to the Natrum-salts, even the Bromine-salt makes no exception. In the *Ammonium*-salts only the two first correspond with the rule, inasmuch as not only the Bromine combination, but also the phosphoric salt are opposed to it. On comparing the *Natrum* and *Kali* salts it appears not only in the mean numbers that the potentiating effect at the latter is proportionately greater than in the first, but also in the single ones—*e. g., Iod-Kalium* with —2 in the 3d potency should according to the rule show a weaker total effect than kitchen-salt with —5 in the 3d potency, but it is the reverse: 87 against 76. *Brom-Natrium* with —35 and *Kali-carbon.* with —37 should have an approximately equal total effect, but the first has 112, the latter 127. Of the two Ammonium-salts following the rule, only Ammonium-muriaticum with —34 in the 3d potency is open to comparison, and here the total-effect-number 114 corresponds very nearly with that of Brom-natrium (112). If now we give another construction to the rule expressed in these numbers, it would be: for the potentiating homœopath the degree of poisonousness of a substance in poisonous concentration is *gener-*

*ally no reason* to exclude a substance from the list of applicable medicines, for in a sufficiently high potency this difference disappears more and more, and even the most poisonous substances gain sufficient animating power to functionate as remedies. Now nobody will be able to contend that this numerical result of my Neural Analysis does not correspond exactly with the condition of practical Homœopathy, which hesitates not a moment to administer the strongest poisons as remedies just as well as it operates with such harmless substances as kitchen-salt, Sulphur, Carbon, etc. But : also this taught the neuro-analytical numbers : *nulla regula sine exceptione!* Whilst of the 6 Natrum-salts the 25th potency appears with numbers between +69 and +87 of 3 Kali-salts with +85 — +90, of 2 Ammonium-salts with +80 and +84, *Brom-kalium* and *Ammonium-phosphor.* place themselves as *exception* on the opposite side with the numbers +43 in the 25th potency. These two substances defend their severe paralytic power in the 3d potency with the numbers —70 and —64 with an obstinacy as no other (Brom-Ammonium excepted which is already disposed of). Not only up to the point of indifference, but also in the higher potencies it shows itself by a slower increase of the potentiating effect. A conclusive judgment on this point can evidently only be obtained by a duration-measurement on the deglutition method, as was done with Kali-carbonicum. I am convinced that on extension of these investigations of further groups of substances enough such obstinate substances will be met, and I have already given hints in this direction when speaking of Brom-Ammonium.

(*f.*) Now we return once more from the *exception* to the *rule.* The fact is that not only Homœopathy without hesitation resorts to the most violent poisons, but many practitioners say that the *most violent poisons* become the *most powerful medicines* by the process of attenuation. Also this view can be sufficiently confirmed by the numerical material—*First,* by the difference between the *Natrum*-salts on one hand and the *Kali*-salts on the other hand. That the Kali-salts are more poisonous than the Natrum-salts is the common result of neural analysis and other experience, and aside of two exceptions the 25th potency has at the first higher animating effects than at the latter (85, 90, 89 with Kali-salts, 69, 70, 71, 71, 77 with Natrum-salts). *Second.* Next, the Brom-Ammonium, already spoken of among the Natrum-salts, the phosphoric Natrum with —25 in 3d potency has the greatest poisonousness and its 25th potency rises with +87 beyond the animating value of all the others. *Third.* After the Natrum-salts the *Sulphuric* salt with —19 in its 3d potency approaches in poisonousness nearest to the phosphoric salt; to this corresponds that its 25th potency with +77 according to the number of the phosphoric salt is the highest among the Natrum-salts. *Fourth.* Also at the Kali-salts we find proofs: The weakly poisonous Iod-Kalium has in the 25th potency with +85 less animating effect than the more poisonous phosphoric and carbonic salts with +89 and +90. *Fifth.* Among the Ammonium-salts the carbonic and hydrochloric salt stand in equal proportion; the more poisonous first has in the 25th potency +84, the less poisonous latter only +80. Therefore, also in this respect the numerical result harmonizes with views which owe their origin to practical experience, and this also may be considered as a correspondence that only rules are concerned of which there are exceptions, and not commonly valid pattterns.

"Well, if that is so, then Neural Analysis, properly speaking, brings nothing new!"

That it shall and will not bring; what it will and can is that in the place of more or less vague opinions and doubtful assertions brings "security, certainty," instead of words "numbers," and that it removes Homœopathy out of the obscurity of mysticism, from the department of pure empiricism to the clear light of the exact—*i. e.*, measuring and calculating—*science.*

As conclusion remains for this chapter of numerical material, the construction of curves from the numbers for the better illustration.

Table No. VIII. Curves for eight neutral salts. (See original *A. H.* 2, p. 39.)

In the foregoing table of curves I have given only 8 of the measured 14 salts, for at the present scale all 14 salt lines could not well be represented without destroying the lucidity. In this limit the curves of each of the seven salts, the names of which stand at the left side, can be clearly followed. The greater clearness also necessitated, as in the curves of the Kali-carbon, to simplify the net of lines inasmuch as at the vertical potency lines as well as at the horizontal lines the value of the effects was left out of every other line.

The thick horizontal line marked O is *the line of indifference*, *beyond* it lies the scale of the animating effects, provided with plus-signs; *below* it the scale of the paralytic effects, provided with minus-signs. The horizontal series of numbers at the top of the table gives the scale of the decimal potencies.

The curves of the three represented Natrum-salts are drawn with *uninterrupted* lines, and for greater clearness the curve of the *saltpeter*, which runs very near to that of the *kitchen-salt* is distinguished by a thin line, different from the kitchen-salt line. For the curves of the three Kali-salts a line of *small dashes* was used, and the line of the phosphorus-salt was made more conspicuous by inserting points between the dashes. For the two Ammonium-salts *crosses* were used; for the Bromine-salts only crosses; for the phosphorus-salt alternating crosses and points. The curve of the latter salt begins on the table at the 8th potency in the interest of clearness, for from 3 to 8 it crosses the next curve so frequently that the picture would have become confused.

I do not want to tire the reader by repeating the example of the curve of what I communicated to him on reading the table. I only want to draw the attention to what the curve shows more distinctly than the table, viz.: that *every* curve shows *a peculiar specific rhythm of its course*, and that in this the similitude, as well as the specific differences, find expression. Surprising, for instance, is the peculiar corresponding irregularity of the curves of the two Ammonium-salts in opposition to the corresponding regularity of the curves of Natrum-mur. and Nitr. It is also seen easily that the curves of the salts which have equal or similar acids (resp., halogens) are more similar to one another than when acid and base are different. This appears clearly at the Bromine-salts and the phosphates. These appearances of the curves would naturally be observed in much greater extent and distinctness if all the 14 salts had been represented graphically, and like with the Kali-salts; also, the lower half of the three relegated salts had been inserted. As said before, they, after ascertaining the indifference, were set back for the reason that their numbers so nearly coincided with those of Kali-carb. Still, I have for myself drawn the curves of the four so similar Kali-salts: they run so near together, in such corresponding rhythm, and yet through each other so individually that even in double as great a proportion as the above table of curves, the single lines cannot be followed clearly without the help of different colors. The numbers of table III enable the reader to do as I did, if he has a taste for it, the picture will startle him, as it did me at the time.

Herewith I conclude the communication of the numerical material which the labor of the past winter has furnished for potentiation, and in the following sections the communications of the futher physiological phenomena are added, which I had an opportunity to observe at the mentioned work. These are of no less interest and proof for the doctrine of potentiation, than the numbers obtained; practically they are, perhaps, even more important than these, at least at the present state of the matter.

[TO BE CONTINUED.]

